# Healing Field: Using Alternating Electric Fields to Prevent Cytokine Storm by Suppressing Clonal Expansion of the Activated Lymphocytes in the Blood Sample of the COVID-19 Patients

**DOI:** 10.3389/fbioe.2022.850571

**Published:** 2022-06-02

**Authors:** Hamed Abadijoo, Mohammad Ali Khayamian, Mahsa Faramarzpour, Mohammadreza Ghaderinia, Hossein Simaee, Shahriar Shalileh, Seyed Mojtaba Yazdanparast, Bahman Ghabraie, Jalil Makarem, Ramin Sarrami-Forooshani, Mohammad Abdolahad

**Affiliations:** ^1^ Nano Electronic Center of Excellence, Nano Bio Electronic Devices Lab, School of Electrical and Computer Engineering, University of Tehran, Tehran, Iran; ^2^ Nano Electronic Center of Excellence, Thin Film and Nano Electronics Lab, School of Electrical and Computer Engineering, University of Tehran, Tehran, Iran; ^3^ Institute of Cancer, Imam Khomeini Hospital, Tehran University of Medical Sciences, Tehran, Iran; ^4^ UT and TUMS Cancer Electronics Research Center, Tehran University of Medical Sciences, Tehran, Iran; ^5^ ATMP Department, Breast Cancer Research Center, Motamed Cancer Institute, ACECR, Tehran, Iran; ^6^ Department of Experimental Immunology, Amsterdam Infection and Immunity Institute, Amsterdam University Medical Centers, University of Amsterdam, Amsterdam, Netherlands

**Keywords:** COVID-19, cytokine storm, clonal expansion, lymphocyte, alternating electric fields, mitosis suppression, inflammation

## Abstract

In the case of the COVID-19 early diagnosis, numerous tech innovations have been introduced, and many are currently employed worldwide. But, all of the medical procedures for the treatment of this disease, up to now, are just limited to chemical drugs. All of the scientists believe that the major challenge toward the mortality of the COVID-19 patients is the out-of-control immune system activation and the subsequent cytokine production. During this process, the adaptive immune system is highly activated, and many of the lymphocytes start to clonally expand; hence many cytokines are also released. So, any attempt to harness this cytokine storm and calm down the immune outrage is appreciated. While the battleground for the immune hyperactivation is the lung ambient of the infected patients, the only medical treatment for suppressing the hypercytokinemia is based on the immunosuppressor drugs that systemically dampen the immunity with many unavoidable side effects. Here, we applied the alternating electric field to suppress the expansion of the highly activated lymphocytes, and by reducing the number of the renewed cells, the produced cytokines were also decreased. Applying this method to the blood of the COVID-19 patients *in vitro* showed ∼33% reduction in the average concentration of the three main cytokines after 4 days of stimulation. This method could carefully be utilized to locally suppress the hyperactivated immune cells in the lung of the COVID-19 patients without any need for systemic suppression of the immune system by the chemical drugs.

## Graphical Abstract

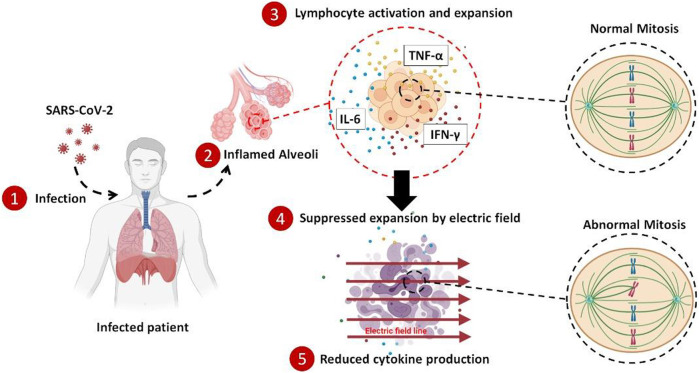

## Introduction

More than 200 million infections with at least four million mortalities (World Health Organization, 2021) worldwide are just a small part of the consequences that a 100 nm coronavirus (Zhu et al., 2020) has imposed on the world community since its emergence in China in 2019 (Phelan et al., 2020). After infection and commencement of the disease’s immunological phase, many clinical manifestations would arise, but acute respiratory distress syndrome (ARDS) is the most lethal feature of the COVID-19 (Gibson et al., 2020; Ragab et al., 2020). Numerous pieces of evidence suggest that the severity of the disease and ARDS is highly correlated with the proinflammatory cytokine levels in the bloodstream and the intensity of the immune cell hyperactivation caused by the virus (Mangalmurti and Hunter, 2020; Mehta et al., 2020; Vaninov, 2020; Khayamian et al., 2021).

Out-of-control secretion of cytokine proteins with subsequent immune system hyper-activation causes severe systemic damages to the tissues and organs throughout the body, such as pulmonary dysfunction and renal failure (Bhaskar et al., 2020; Rabb, 2020; Sun et al., 2020). To prevent exacerbation and progression of the disease in such patients, a broad range of immunosuppressor drugs such as corticosteroids, JAK inhibitors, etc., are used as a general treatment (Abdin et al., 2020; Kolilekas et al., 2020; Sharun et al., 2020; Ye et al., 2020; Hu et al., 2021). For instance, dexamethasone therapy has shown promising results in reducing the severity of the infection and suppressing cytokine storm and the consequent hyperinflammation phase of the disease (Chen et al., 2006; Lammers et al., 2020; Group et al., 2021; Noreen et al., 2021). One major effect of the immunosuppressor drugs such as dexamethasone is their antiproliferative effect. In fact, dexamethasone impairs the proliferation of the lymphocytes such as T cells during their clonal expansion (Creed et al., 2009; Maślanka, 2013; Maślanka and Jaroszewski, 2013; Giles et al., 2018). Although many benefits are associated with the administration of dexamethasone for suppression of severe inflammations, many serious side effects (Alessi et al., 2020; Langarizadeh et al., 2021), such as the increased risk of sepsis (Bendel et al., 2002; Pera et al., 2002), calcium metabolism (Caniggia et al., 1981; Kusuda et al., 2019), kidney disorders (McKay and Cidlowski, 2003; de Vries et al., 2010), etc., (Russell et al., 2020; Hu et al., 2021), are also inevitable.

Cytokines, as the signaling proteins and mediators of the immune response to the inflammation, are secreted by a variety of the cells, including lymphocytes, granulocytes, macrophages, endothelial cells, fibroblasts, etc., among which T cell lymphocytes are the dominant agents that level up the released cytokines in the bloodstream (Zhang and An, 2007). Part of the cytokine secretion by the lymphocytes is attributed to their clonal expansion, a phenomenon by which activated lymphocytes produce more of themselves through mitosis division with the same antigen against a specific pathogen (Condotta and Richer, 2017; Adams et al., 2020; Gutierrez et al., 2020; Liao et al., 2020). Hence, controlling such mitosis would be of interest in the suppression of immune hyperactivation in COVID-19 patients.

Alternating electric field (AEF) therapy, mostly called tumor-treating fields (TTF), is a safe treatment of highly proliferative cancer cells by delivering the low intensity and intermediate frequency electric field (Carrieri et al., 1991; Hottinger et al., 2016). The method disrupts the mitotic spindle assembly of the dividing cells (Kirson et al., 2007). The abnormally divided cells with prolonged mitosis phase (during their cell cycle) finally undergo apoptosis. Since the AEF has an antimitotic mechanism, the most affected cells are highly proliferative ones, such as rapid growing cancer cells, while the healthy cells with a low division rate are not damaged during this stimulation (Kirson et al., 2004; Giladi et al., 2015).

On this basis, AEF could safely be applied for each patient to suppress the highly activated and expanding lymphocytes and consequently reduce the amount of the released cytokines into the blood. As previously mentioned, such a procedure is currently implemented using the systemic administration of the immunosuppressor drugs that debilitate the entire immune system (Mehta et al., 2020). AEF stimulation just impacts the highly expanding lymphocytes in the lung ambient without any effect on the other immune cells, contrary to the immunosuppressor drugs that impair all of the immune cells.

Here, we investigated the possibility of using the AEF modality to suppress the clonal expansion of highly activated and proliferative lymphocytes and the consequent reduction in the cytokines released by these cells, suitable as an anti-inflammation technique for COVID-19 patients. For this purpose, the peripheral blood mononuclear cells (PBMCs) were isolated from healthy donors, followed by artificially being activated using the lymphocyte expansion kits. Then, the effect of the AEF on suppressing the expansion of the lymphocytes was evaluated by time-lapse imaging, viability, and apoptosis assays as well as flow cytometry technique. Moreover, the amount of three major cytokines (TNF-alpha, INF- gamma, IL-6) after AEF stimulation was also measured using the ELISA method. Then, the proposed method was verified by *in vitro* stimulating the WBCs derived from the COVID-19 patients with severe inflammation and cytokine storm. Based on the results, an average of ∼33% in cytokine reduction was achieved after 1 week of AEF stimulation on the immune cells of the blood. In the case of artificially activated immune cells by lymphocyte expansion kits, this number was about ∼26%. We believe that the proposed electrical method with promising results by local suppression of the cytokine storm could be a safe substitute for the immunosuppressor drugs with their systemic effects.

## Materials and Methods

### Alternating Electric Field Stimulation Set-Up

The glass substrate was cleaned using piranha solution (H_2_SO_4_: H_2_O_2_ with a volume ratio of 2:1, respectively). Then the substrate was deposited with Cr and Au layers using RF sputtering procedure. The 20 nm Cr layer was used in order to enhance the 100 nm Au layer adhesion to the glass substrate. Using standard photolithography (Jahangiri et al., 2019; 2020), the eight electrodes were patterned on the surface of Au coated glass. The patterned electrodes were passivated using a 4 µm layer of PDMS by spin coating at 6000 rpm for 5 min. The biochip contains eight electrodes designed in a circular pattern with an inner radius of 2 cm to attain symmetrical electric field distribution in four directions of electric field stimulation. Finally, the biochip was inserted into the set-up and connected to the RF function generator for AEF stimulation (100 kHz sinusoid of 3 V/cm electric field amplitude).

### Electric Field Simulation

Potential and electric field distributions were studied using electrostatics physics of COMSOL Multiphysics 5.5 to investigate the electric field distribution in the biochip. The applied electric field was calibrated to obtain the highest area percentage covered by the electric field intensity in the range of AEF stimulation.

### Peripheral Blood Mononuclear Cells Activation and Expansion Protocol

Human PBMC isolation was performed using the density gradient centrifugation method. The isolated PBMCs were diluted to 10^6^ cells/ml in Dulbecco’s Modified Eagle Medium (Cat number: 10566016, Gibco, United States) containing 10% fetal bovine serum (Cat number: 10082139, Gibco, United States) and 1% penicillin-streptomycin (Cat number: 15070063, Gibco, United States). 10^6^ cells were seeded in 6-well plates per well. 3 mls of the cell culture medium were added to each well. To obtain the lymphocyte activation, Human T cell activator (Cat number: 10,970, Stemcell Technologies, Canada) and B cell expansion kit (Cat number: 100-0645, Stemcell Technologies, Canada) were added to the culture medium with concentrations of 25 μl/ml and 20 μl/ml respectively. The cells were incubated at 37°C and 5% CO_2_ in a humidified incubator for 5 days.

### Peripheral Blood Mononuclear Cells Isolation

PBMCs were isolated from freshly drawn blood stored in Heparin capped tubes for 15 min at 37°C. At first, the blood was diluted using PBS (1:1) and then gently layered over Ficoll (Cat number: F4375, Merck, Germany) (4:3) in a centrifuge tube. The whole sample was then centrifuged for 20 min at 2000 rpm. After this process, four layers were formed. The uppermost layer contained diluted blood plasma, and the second layer contained PBMCs. This layer was then gently removed and was added to fresh PBS (1:3) for another two rounds of centrifugation for 10 min at 2000 and 1500 rpm to wash out any remained platelets. Then the PBMCs were diluted in 1 cc of PBS and counted using Trypan blue staining in the Neubauer chamber.

### Immunofluorescence Microscopy

CD8 and CD19 expression in PBMCs was assessed using inverted fluorescence microscopy. As previously reported by our group (Ansaryan et al., 2019; Khayamian et al., 2019), the PBMCs were first fixed in 3.7% formaldehyde for 15 min and permeabilized with Triton X-100 in PBS for 5–10 min (with a concentration of 1%) at room temperature. The cells were then washed with PBS and then treated with a blocking buffer (1% BSA in PBS) for 40 min at room temperature. Then, all samples were washed and stained with the Anti-CD8 Antibody (ab217344-abcam) as the primary antibody, followed by incubation for 18 h, and again stained with Goat anti-rabbit secondary antibody conjugated with Alexa Fluor 488 (A21424-Invitrogen) for 4 h. The samples were also stained for CD19 (ab134114-abcam) with the same procedure. For Acridine Orange (AO), Propidium Iodide (PI), and DAPI staining, the cells were first incubated in the desired dye for 10 min and then washed 2 times using PBS.

### Apoptosis Assay by Flow Cytometry Analysis

Apoptosis assay was measured using Annexin V-FITC Apoptosis Detection Kit (ab14085, Abcam, Cambridge, United Kingdom). After AEF stimulation of each sample, PBMCs were collected by centrifugation and resuspended in 500 μl of binding buffer. After this process, 5 μl of Annexin V-FITC and 5 μl of Propidium Iodide (PI 50 μg/ml) were added to the samples, and after incubation at room temperature for 5 min dark, the fluorescent intensity was measured by the Flow cytometry (FACScan Becton Dickinson, Mountain View, CA).

The PBMCs were also stained with CD4 Tx red, CD8 APC, and CD19 PE for further analysis using flow cytometry.

### Cytokine Measurement by ELISA

Using Cytokine ELISA Kits allowed to accurately measure the levels of three cytokines including IL-6 (Cat number: D6050, R&D Systems, United States), TNF-α (Cat number: QK210, R&D Systems, United States), and IFN-γ (Cat number: DIF50, R&D Systems, United States) measured with an enzyme-linked immunosorbent assay. Specific concentrations of recombinant human IL-6, TNF-α, and IFN-γ along with the experimental samples were added and incubated in polystyrene microtiter plates coated with an antibody against the appointed cytokine, then incubated with an enzyme-linked polyclonal antibody directed to the cytokine. In the next step, a substrate solution for the enzyme was added, and the color development was stopped by adding 2N H2SO4. The absorbance was measured with a microplate spectrophotometer. The amount of IL-6, TNF-α, and IFN-γ in each sample was measured through a standard curve generated in each assay and expressed as picograms per milliliter. The sensitivity of the enzyme-linked immunosorbent assay for IL-6, TNF-α, and INF-γ is 0.70 pg/ml, 1.88 pg/ml, and 8 pg/ml, respectively. The reproducibility of all measurements was within 10% in our laboratory.

### Blood Sampling From COVID-19 Patients

The blood samples were drawn from covid-19 patients who signed the written consent.

### Ethics Statement

Each and every blood sample used in this research was obtained from donors who signed the written consent. Consents were obtained for all participants conscious of the planned experiment and the subsequent publication and all of the experiments were conducted following relevant guidelines.

### Statistical Methods and Data Analysis

All the collected data were analyzed by GraphPad Prism software version 8.3.0 (GraphPad Software, Inc. La Jolla, CA, United States), and each data point represents the mean value of three independent measurements.

## Results

### Alternating Electric Field Stimulation Set-Up and Electric Field Simulation

**FIGURE 1:**
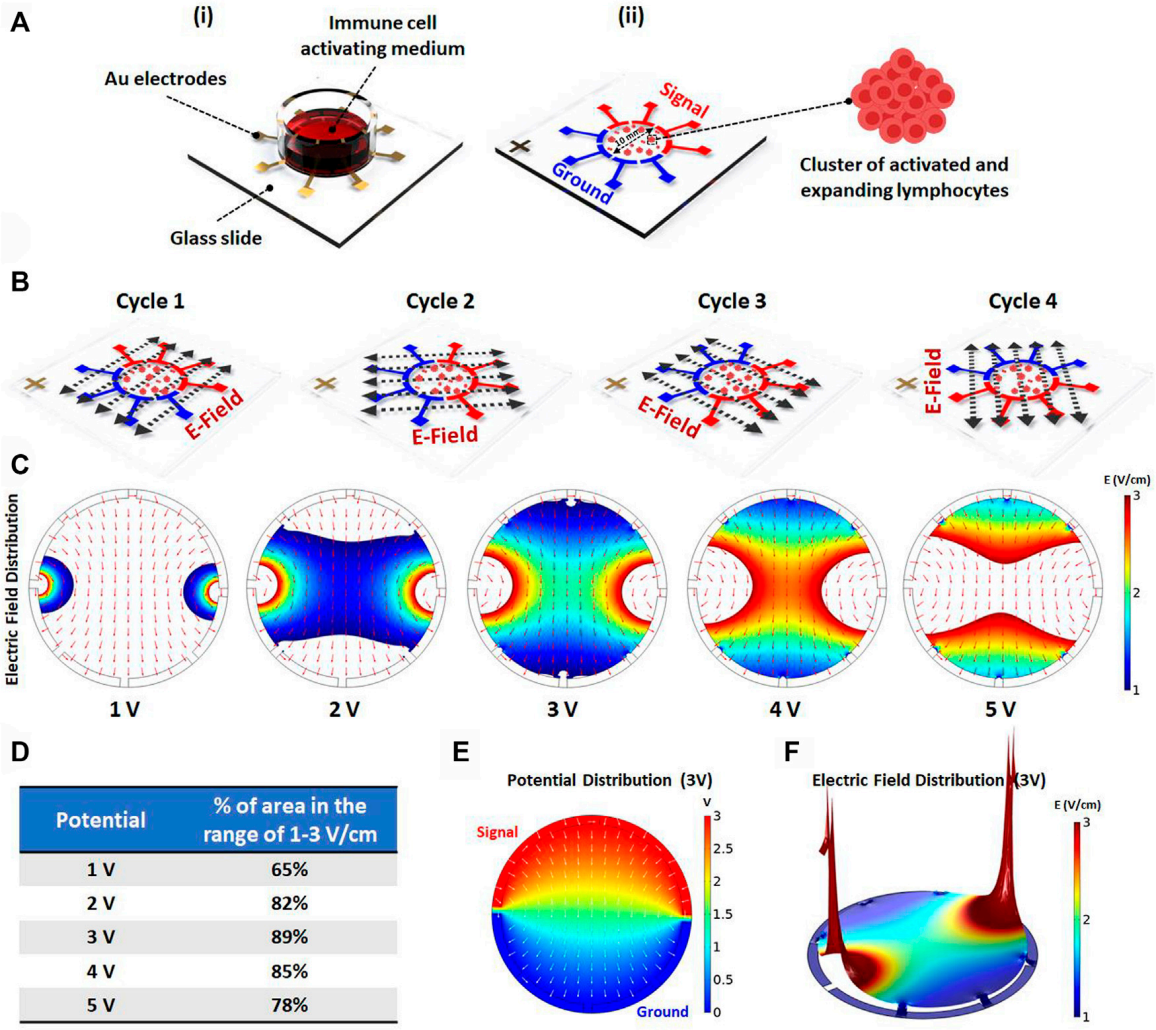
**(A**_**i**_ **)** Schematics showing the electric field stimulation set-up. The set-up structure consists of Au patterned electrodes on a glass slide to generate alternating electric fields in four directions. The PBMCs are cultured inside the immune cell activation medium, which is placed on Au patterned electrodes. **(A**_**ii**_ **)** The 100 kHz alternating voltage is applied to two groups of electrodes (each group containing four electrodes) in order to obtain uniform electric field distribution inside the immune-cell activation medium containing clusters of activated and expanding lymphocytes. **(B)** The electric field stimulation system was designed to periodically change the electric field direction in four cycles (each cycle lasts for one second) to increase the chance of parallelism between the electric field direction and the axis of cell division. **(C)** The electric field distribution inside the immune cell activation medium was studied using COMSOL Multiphysics AC/DC module. The simulation was performed for different applied voltages to evaluate the area percentage stimulated with preferred electric field intensity (1–3 V/cm) for each voltage. **(D)** The area percentage stimulated with preferred electric field intensity was quantified for each voltage. The simulation demonstrates that the highest area percentage is obtained by 3 (Volts) stimulation voltage amplitude. **(E)** The surface plot shows the electric potential distribution corresponding to the optimum applied voltage. The normalized arrow surface plot demonstrates the electric field directional distribution. **(F)** The color range-limited 3D plot illustrates the electric field intensity distribution inside the activation medium corresponding to the optimum applied voltage. (*p < 0.05, **p < 0.01, ***p < 0.001, and ****p < 0.0001, t-test).

To investigate the effect of AEF on the expansion of the activated lymphocytes, an array of eight electrodes in a circular shape were patterned on top of a glass slide (Figure 1A_i_). The cells were cultured on the center of the stimulating chip, and the electric field was applied by the use of surrounding electrodes. For better uniformity of the electric field distribution all over the chamber, each pole consists of four electrodes with a face-to-face array and in a symmetrical pattern (Figure 1A_ii_).

Mitotic arrest induced by the AEF only occurs for the cells whose mitotic spindle is aligned in the electric field direction, while the cells with unaligned spindle would not be affected. Therefore, to impact all of the expanding cells with different spindle alignment, the signal (sine wave with an amplitude of 3 V and frequency of 100 kHz) is switched every second (0.25 Hz) between the electrodes to apply the electric field in all of the directions (Figure 1B).

Based on works of literature, the safe and effective intensity of AEF stimulation for suppressing the mitosis of the proliferative cells is in the range of 1–3 V/cm (Giladi et al., 2015). Hence, the applying voltage was optimized in a way that most of the cells experience the electric field in the mentioned range. For this purpose, the field intensity over the surface of the chip was calculated using the AC/DC physics of COMSOL Multiphysics simulation software. The applied voltage to the electrodes (Figure 1C) was swept in different ranges to extract the optimized electric field. Based on the results (Figure 1D), the electric potential of 3 V was selected as the optimum applied voltage because the most cell seeding area was observed in the range of 1–3 V/cm. Figures 1E,F demonstrate the electric potential and 3D electric field distribution for the applied voltage of 3 V.

### Mimicking the Clonal Expansion by Activating the Peripheral Blood Mononuclear Cells

Lymphocytes are one of the subsets of white blood cells with mitosis capability (Oliaro et al., 2010). When these cells are activated by external physicochemical signals, they form a cluster of cells and then are expanded (Jevnikar et al., 2008; Zumwalde et al., 2013; Cheung et al., 2018). In the case of T and B lymphocytes, such a phenomenon is called clonal expansion in which the first activated cell produces many copies of itself with the same antigen properties (Adams et al., 2020).

**FIGURE 2:**
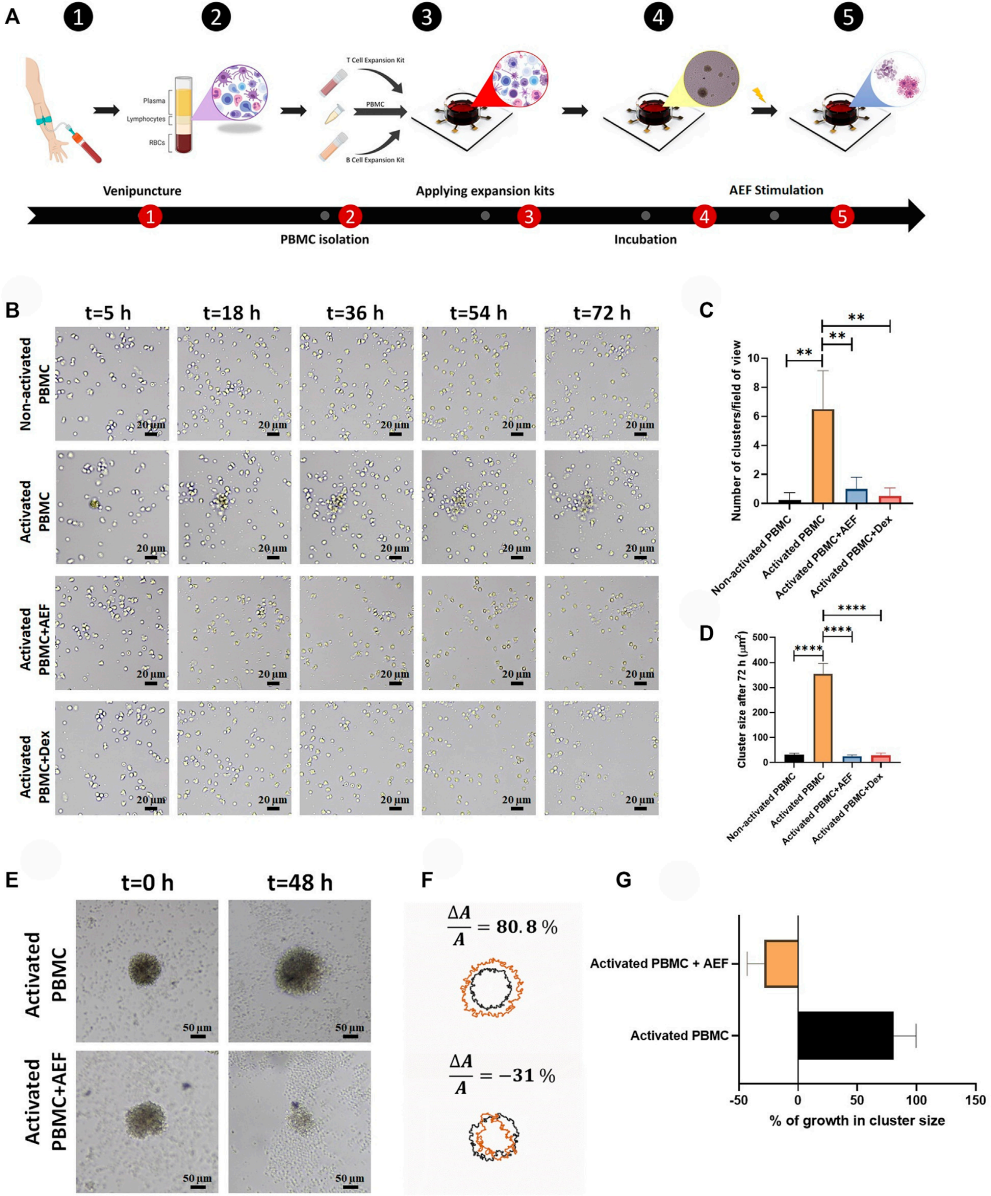
**(A)** The whole blood was obtained through the venipuncture technique. The PBMCs were isolated from the whole blood using density gradient centrifugation. Isolated PBMCs were cultured inside the cell culture medium and their lymphocytes were activated by the use of expansion kits. The whole set-up was incubated for 5 days to ensure lymphocytes activation. The system was stimulated using the alternating electric field for 48 h which caused apoptosis in clusters of activated lymphocytes during cell division. **(B)** Lymphocyte cluster formation inside the immune cell activation medium was evaluated *via* time-lapse imaging. No sign of cluster formation was observed in non-activated PBMCs, while clustering was confirmed in activated PBMCs. Alternating electric field stimulation and the use of dexamethasone (4 mg/ml) similarly inhibited cluster formation in activated PBMCs. **(C, D)** The highest number of clusters were observed in activated PBMCs. These clusters were moderately larger in comparison with clusters formed in three other groups. **(E, F)** Activated lymphocyte clusters were stimulated using the alternating electric field for 48 h, and their size was monitored *via* time-lapse microscopy. The results demonstrated 80.8% growth in the size of activated lymphocyte clusters, whereas the size of the clusters undergoing AEF was reduced by 31%. **(G)** Percentage of growth in cluster size of activated PBMCs in control and AEF stimulated groups. (*p < 0.05, **p < 0.01, ***p < 0.001, and ****p < 0.0001, t-test).

In order to mimic the expansion of the WBCs, the T and B activation kits (Cat number: 10,970, Cat number: 100-0645, Stemcell Technologies, Canada) were utilized to artificially trigger the expansion of the lymphocytes in the PBMCs of blood *in vitro*. As could be deduced from Figure 2A, 4 days after PBMC activation, many of the clusters were formed in different sizes with increasing rates by the time. The bigger size of the cluster demonstrates the greater number of activated and expanding lymphocytes.

### Time-Lapse Imaging to Investigate the Effect of Alternating Electric Field on Activated-Peripheral Blood Mononuclear Cells

Time-lapse imaging was used from the PBMCs at different times to investigate the ability of the AEF stimulation to suppress the proliferation of the activated lymphocytes. For this purpose, the PBMCs were isolated from the normal donors by density gradient centrifugation (DGC) method and then divided into four groups: 1) Non-activated PBMC--without any external stimulation (intact WBCs are imaged as the control group), 2) Activated PBMC--the WBCs are begun to expand by the commercial activating kits for T and B cells, 3) Activated PBMC + AEF and 4) Activated PBMC + Dexamethasone (4 mg/ml). In this regard, the effect of AEF in suppressing clonal expansion would also be compared to dexamethasone (conventional immunosuppressor drug).

As shown in Figure 2B, in the case of intact WBCs (Non-activated PBMC), no cluster formation could be seen after 72 h as all cells are moving alone. But, in the group of activated PBMCs, small immune cell aggregations could be tracked, rapidly growing in colony size and covering the whole surface after 20 h confirming the clonal expansion of the activated lymphocytes (Figures 2C, D).

Interestingly, when the cells are simultaneously treated with the alternating electric field (AEF), no growth of the cell clusters could be seen, which shows that even the activated cells were not able to be expanded. Such antiproliferative immunosuppressing result could also be tracked in the Activated PBMC + Dexamethasone group. This outcome corroborates that both AEF and Dexamethasone have a similar function in suppressing the mitosis process of the activated lymphocytes.

For more elaboration, the AEF stimulation was applied on the activated immune cells after 5 days. During these 5 days, the clonal expansion clusters of immune cells were formed Figure 2E. Then, cluster size and their abundance were imaged and analyzed after 48 h of AEF treatment. Contrary to the continuous growth of cell clusters in the cohort of activated PBMC, the proliferation and expansion of the activated PBMC were strongly suppressed by the AEF stimulation. As presented in Figures 2F,G, the average cluster size shows an increase of about ∼81% after 48 h for the non-AEF treated group, while the growth of clusters in the electrically stimulated group has been dropped to ∼ −31%. These results confirm that the suppressive effect of AEF stimulation, which had been confirmed on the proliferative cancer cells (Giladi et al., 2015; Kessler et al., 2018), is also applicable for the expanding activated lymphocytes.

### Viability of the Stimulated Clusters and the Non-activated WBCs

**FIGURE 3:**
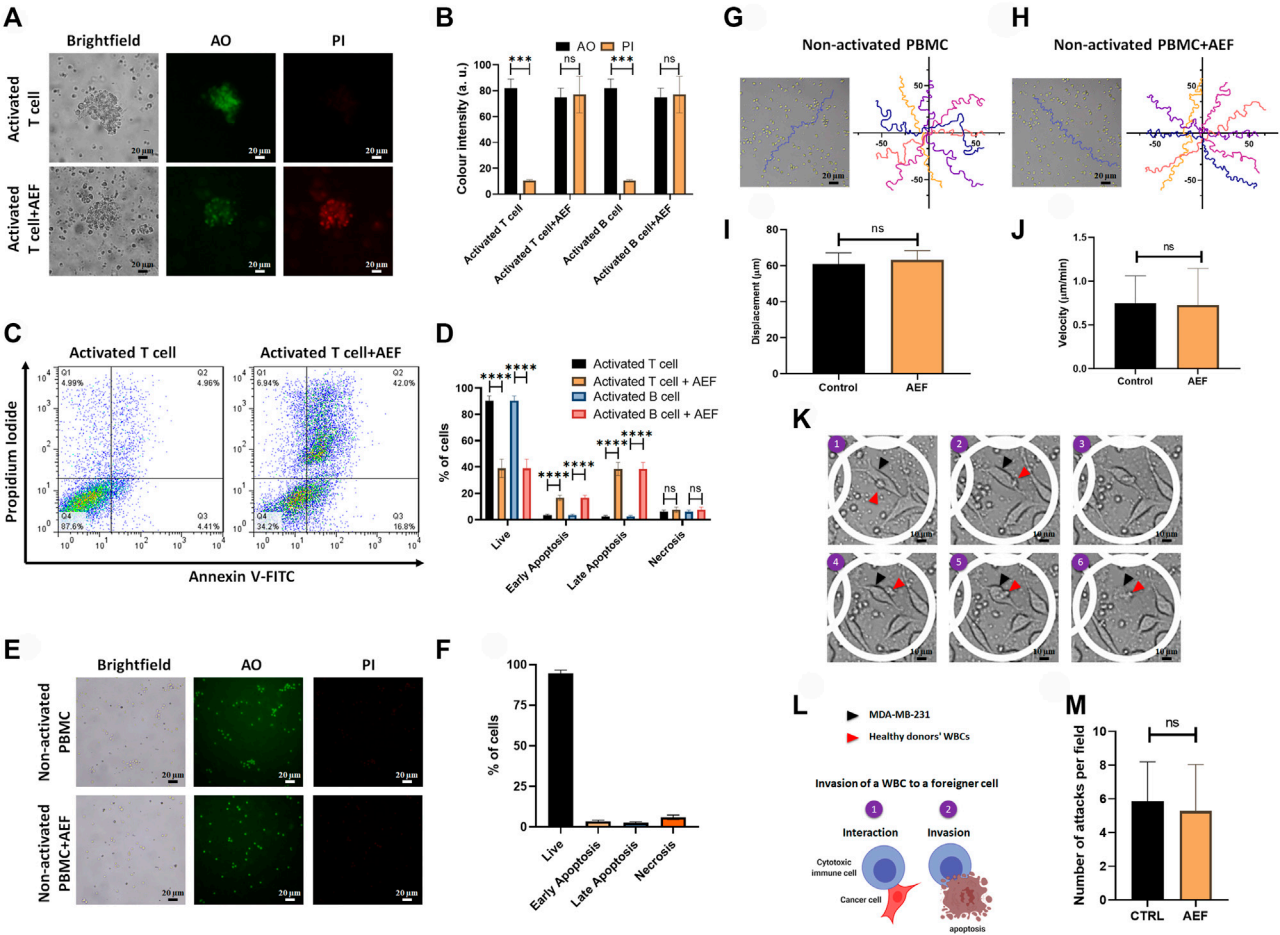
**(A, B)** Immunofluorescence imaging shows elevation of PI uptake in activated lymphocytes by 48 h of AEF stimulation. PI uptake in the control group is negligible. **(C, D)** Annexin V/PI assay demonstrated the increase in the percentage of activated lymphocytes in the apoptosis phase caused by AEF. A negligible difference was observed in cells undergoing necrosis in both groups. **(E, F)** Immunofluorescence imaging demonstrates negligible PI uptake in both groups of control and AEF-stimulated non-activated PBMCs due to the effectiveness of AEF on dividing cells. **(G, H)** Cell trajectories were plotted for non-activated PBMCs in control and AEF-stimulated groups. **(I, J)** No significant difference was observed between displacements and velocities in these two groups. **(K)** Time-lapse imaging on the interaction of the electrically stimulated WBCs from a human donor with MDA-MB-231 cancer cell line. **(L)** Schematic representation of the WBC invasion to a foreigner MDA-MB-231 cell **(M)** Number of attacks by the WBCs to the foreigner MDA-MB-231 cells for the control and stimulated WBCs. (*p < 0.05, **p < 0.01, ***p < 0.001, and ****p < 0.0001, t-test).

AO/PI staining as well as apoptosis assay by Annexin V/PI flow cytometry technique was carried out to find the correlation between the cellular viability and the size reduction of the treated clusters. For more clarification, the viability and apoptosis assays were performed separately on lymphocytes, including B and T cells. Based on the image analysis, all of the two WBC subsets after AEF stimulation show a noticeable increase in the expression of the PI dye, which is an indicator of the membrane rupture and cell death (Khayamian et al., 2018) (Figures 3A,B). No PI uptake could be tracked in the control groups representing the viability of the cells. In harmony with the results of the AO/PI, flow cytometry results also show a significant reduction in the fraction of the live cells for the AEF treated group in which most of the cells entered into the early and late apoptosis phase (Figures 3C,D). As presented in Figure 3D, it is noteworthy to say that the necrosis portion of the cells is roughly the same before and after the electric field treatment. This phenomenon corroborates the non-necrosis induction of AEF on the expanding immune cells because based on the AEF mechanism, the cells undergo apoptosis, and sudden death or necrosis does not happen.

In the next step of the study, the effect of the AEF on the non-activated white blood cells was evaluated by live/dead staining and apoptosis assay. There is no sign of the cell clustering on the CTRL group since the cells are intact and no activating kit was used (Figure 3E). As presented in Figure 3E, no PI uptake could be traced in the CTRL WBCs before or after the electrical stimulation. This evidence corroborates the fact that the AEF only impacts the proliferative cells. In harmony with the AO/PI assay, the results of the Annexin V/PI test also show no apoptosis in the non-activated immune cells (Figure 3F) after 48 h of AEF treatment. The trajectory of the WBCs (Figures 3G,H) as well as their displacement and velocity (Ghaderinia et al., 2021) were also analyzed for non-activated WBCs in the presence and absence of AEF. As shown in Figures 3I,J, no difference could be inferred in their natural moving behavior. For further clarification, the effect of the electric field on the invasive function of the WBCs toward pathological threats was assessed. Time-lapse imaging (Figure 3K) was utilized to inspect the interaction of the WBCs with the foreigner MDA-MB-231 cells. Due to the HLA mismatch between the MDA-MB-231 cells and the donor WBCs, it is supposed that the cytotoxic T cells (CTLs), as well as natural killer cells (NK cells), are activated and invade the cells (Figure 3L) (Martínez-Lostao et al., 2015; Shenouda et al., 2017; Cerignoli et al., 2018). Based on the results, there is no significant alteration in the number of attacks by the immune cells to the MDA-MB-231 cells, which corroborates the safety of the AEF on the normal function of the immune cells (Figure 3M).

### Details of the Affected Lymphocyte Subsets by Flow Cytometry Analysis

**FIGURE 4:**
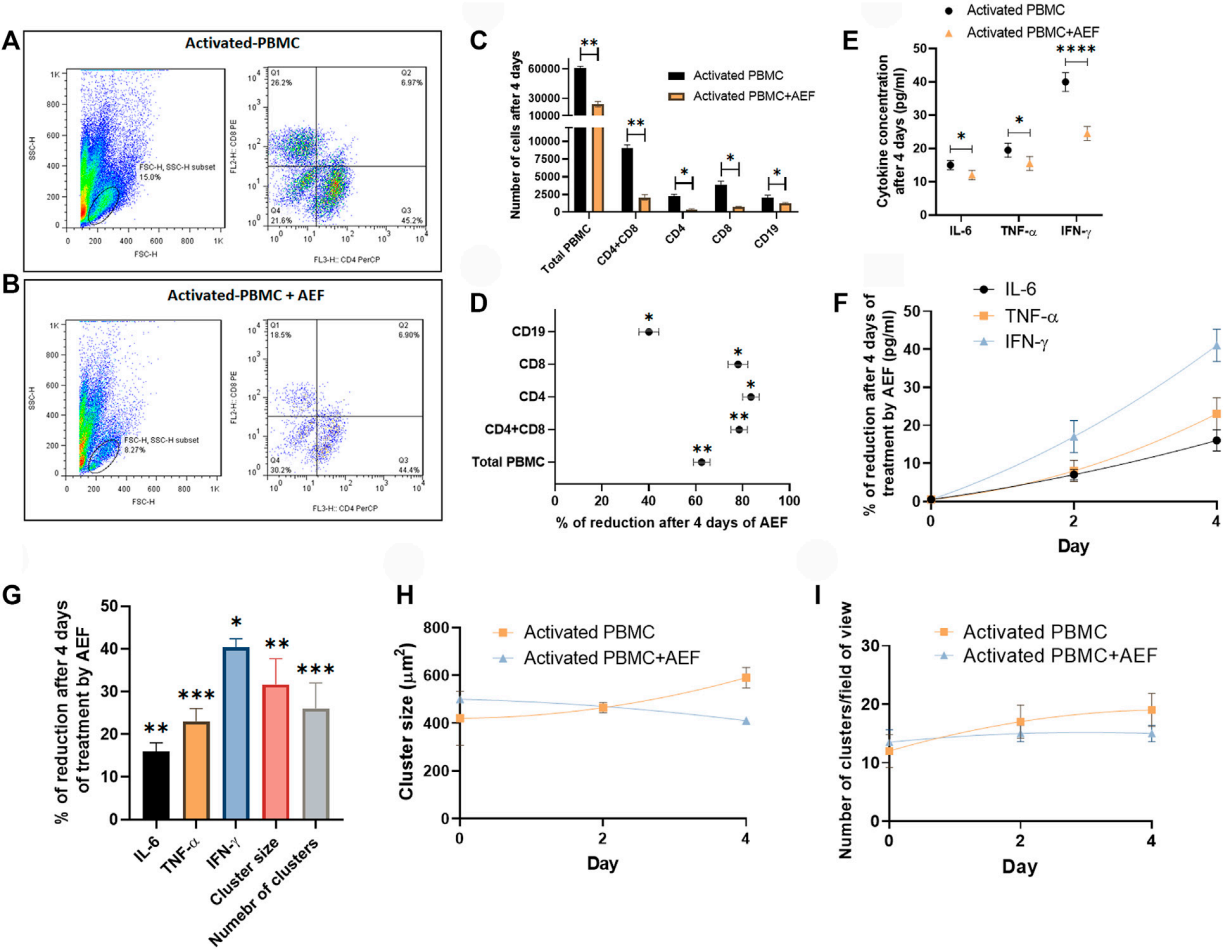
**(A, B)** Flowcytometry analysis confirmed the reduction of activated PBMCs exposed to AEF for 4 days compared to their control group. **(C, D)** Comparison between the number of cells for each group of lymphocytes in PBMC and their percentage of reduction. **(E, F)** The decline in the concentration of inflammatory cytokines in PBMCs caused by AEF stimulation and the daily percentage of reduction for each cytokine. **(G)** Percentage of reduction in the concentration of the inflammatory cytokines, the cluster size and the number of clusters in the activated PBMCs after 4 days of the AEF treatment. **(H, I)** comparison between the daily amount of the cluster size and the number of clusters per field of view for the activated PBMCs in control and AEF-stimulated groups. (*p < 0.05, **p < 0.01, ***p < 0.001, and ****p < 0.0001, t-test).

Flow cytometry technique was employed to investigate the detailed effect of alternating electric field on each subset of the lymphocytes. For this purpose, both of the B and T cell activation and expansion kits were simultaneously applied to the blood of a healthy donor. The cells (after 5 days) were then treated with the AEF stimulation for 4 days, and the number of CD8 and CD4 positive cells (T cell markers) and CD19 cells (B cell marker) were counted by the flow cytometer. The results were compared with the activated but non-AEF treated cells as the control group. Based on the results (Figures 4A,B), the population of all pre-activated lymphocyte subsets was drastically reduced after the AEF treatment (Figure 4C). For instance, the T lymphocytes, including CD8^+^ and CD4^+^ cells, have lost about ∼80% of their population after AEF stimulation and this fraction for B cells is about ∼40% (Figure 4D). The results are in harmony with the expectation that the proliferation of the expanding cells was suppressed by the AEF stimulation. When the activated cells lose their ability to expand and are arrested in the mitosis phase of their cell cycle due to an abnormal division, they enter the apoptosis phase. Therefore, the number of cells in the AEF treated group becomes lower than the control activated and expanding cells. Moreover, T cells, including CD4^+^ and CD8^+^ cells, are more affected by the electric field treatment. This is due to the fact that T cells expand faster than the B cells, and as a result, the AEF stimulation has more suppressing impact on their division.

### Inspecting the Cytokine Reduction After Electric Field Treatment

Finally, the cytokine production by the immune cells was studied in both groups of activated control and AEF-treated cells. The cells at first were activated for 5 days, and then the experiment was carried out for all of the groups. In the activated PBMC + AEF group, the cells were under AEF stimulation for about 4 days. During these 4 days, the non-AEF treated group was just kept in an incubator. At the end of the experiment, the solution medium of both groups was collected and analyzed by the ELISA method.

Although many cytokines are produced and play functional roles in the post-activated immune cells, there are three main cytokines, including IL-6, TNF-α, and IFN-γ that are produced by the lymphocytes and play the main role especially in the COVID-19 disease (Chen et al., 2020; Lammers et al., 2020; McGonagle et al., 2020; Mehta et al., 2020). Based on the results, all three major cytokines have shown a considerable reduction after 4 days of the electric field stimulation (Figures 4E,F). As presented in Figure 4G, the most cytokine reduction was attributed to the IFN-γ with ∼40% and the least for the IL-6 with ∼16% (∼25% for TNF-α). We extremely believe that this decrease is associated with the reduced number of immune cells after the suppression of their proliferation by the AEF. Hence, the number of the clusters, as well as their size, was assessed for the two groups at the end of the study. As shown in Figures 4H,I and in harmony with the results of the cytokine profiling, a reductive trend could be observed in the case of cluster size and abundance by 32 and 26% (Figure 4G), respectively.

### Effect of Alternating Electric Field on Suppressing Clonal Expansion and Cytokine Production in COVID-19 Patients

Human blood samples were collected from the five patients with COVID-19 disease to assess the efficacy of the alternating electric field on suppressing the activation and expansion of immune cells and the consequent reduction in cytokine production. The patients had not received any medication prior to blood sampling. After blood sampling and isolation of the WBCs, the cells were divided into two cohorts of control (without electrical treatment) and the group of electrically stimulated cells. Both groups were under study for 4 days.

**FIGURE 5:**
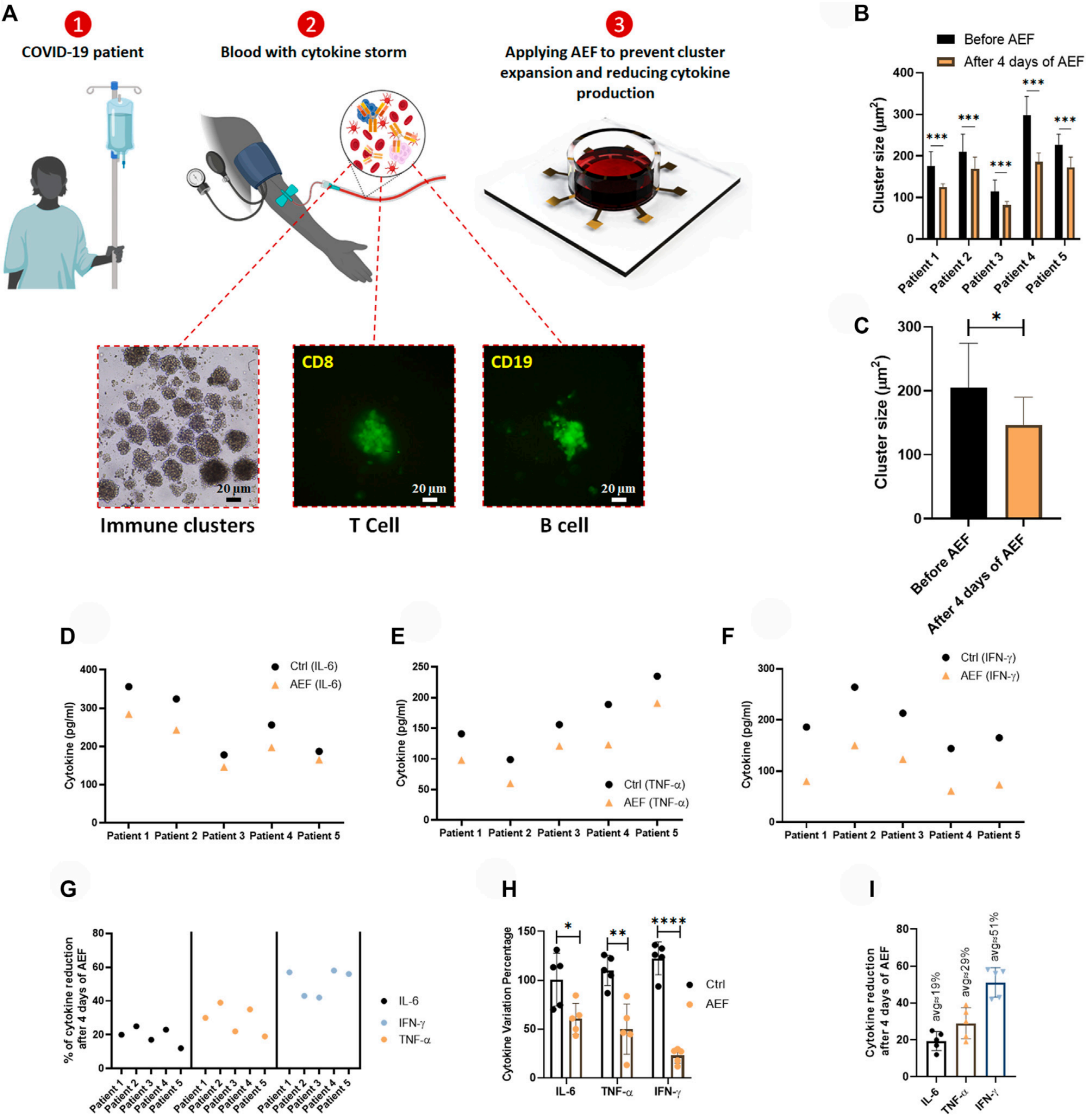
**(A)** The whole blood sample was obtained from COVID-19 patients, and the PBMCs were isolated using the density gradient centrifugation method. Immunofluorescence imaging confirms the activation and the expansion of lymphocytes in these blood samples. The lymphocytes were cultured in blood serums and were stimulated by AEF for 4 days. **(B, C)** Significant differences were observed in the size of lymphocyte clusters after 4 days of AEF stimulation for COVID-19 patients’ PBMCs. **(D–F)** The concentration of inflammatory cytokines in COVID-19 patients’ blood serum, which their PBMCs were cultured, for control and AEF exposed groups. **(G)** Percentage of cytokine reduction in COVID-19 patients’ blood serum caused by AEF stimulation. **(H)** Comparing the cytokine variation percentage for the three cytokines of IL-6, TNF-α and IFN-γ after 4 days of incubation for both groups of control and AEF with respect to the primary sample. **(I)** The average percentage of cytokine reduction in COVID-19 patients’ blood serum after 4 days of exposure to AEF. (*p < 0.05, **p < 0.01, ***p < 0.001, and ****p < 0.0001, t-test).

The time-lapse imaging from the samples of the patients reveals that the lymphocyte clusters are formed (Figure 5A) in the samples of the patients due to cytokine storm. The size of the produced clusters was analyzed after AEF stimulation, and similar to the previous results, the electrical stimulation halted the growth of the clusters in the blood sample of each patient (Figure 5B), and also their average number was also decreased by ∼30% (Figure 5C).

As presented in Figures 5D,G, all of the three major cytokines in the blood sample of all five patients were reduced after 4 days of AEF stimulation, even though the amount of reduction is different for each patient. Also, a major difference in concentration of the all of the three inflammatory cytokines could be observed compared to their initial values (Figure 5H). Figure 5I displays the average amount of cytokine reduction after electric field treatment of the patients’ PBMCs. Based on the results, the most cytokine reduction is related to the IFN-γ by ∼51%, while this number for TNF-α and IL-6 is ∼29 and ∼19%, respectively.

Similar to the previous results obtained by the AEF treatment of the artificially activated PBMCs, we hypothesize that the decrease in the production of the cytokines in the samples of the COVID-19 patients is due to the suppressed clonal expansion of the lymphocytes. In fact, when the lymphocytes are confronted with the SARS-CoV-2 virus antigens, they become activated and then start to expand (Gutierrez et al., 2020). But here, we have suppressed their proliferation by the AEF method as a substitute to the prevalent immunosuppressor drugs such a dexamethasone.

## Discussion

The most challenge attributed to the positive COVID-19 cases that puts the lives of the patients in danger is the hyperactivation of the immune cells and their consequent high cytokine releasement into the bloodstream or the so-called cytokine storm (Huang et al., 2020; Pearce et al., 2020). Currently, the typical remedy for harnessing such an imposed lethal storm is using immunosuppressor drugs, e.g., dexamethasone, which entirely damps the immune system in the body. Although immunosuppressors subside the hyperactivation of the immune system, their various and serious side effects have always been a challenge and limited their prescription. Hence, any other safe and effective treatment that controls cytokine production with less or no side effects is highly appreciated.

Since part of the cytokine storm in the COVID-19 disease is due to the hyperactivation of the lymphocytes and their uncontrolled growth, We, for the first time, have introduced AEF (a known procedure in brain tumor treatment (Fabian et al., 2019)) on electrical stimulation of the lymphocytes to suppress their clonal expansion (Figure 2). Subsequently, the cytokine storm is also reduced due to the reduction of the number of cytokine-producing cells (Figure 4 and Figure 5).

Based on the obtained results (Figure 3), the AEF method had no harmful effects on the viability and function of the non-expanding and non-activated immune cells and other blood cells. The electrical stimulation just perturbs the expansion of the highly proliferating lymphocytes formed as a cluster. This suppression in lymphocyte expansion is followed by a noticeable reduction in the amount of the produced cytokines.

We believe that this method can safely take the place of the current immunosuppressor drugs such as dexamethasone, which is regularly used for cytokine storm treatment that represses the whole immune system all over the body with severe side effects. In the future trend, the electric field could safely be delivered to the lung of the patients to locally suppress the highly proliferating cytokine-producing lymphocytes as a complementary method.

## Data Availability

The original contributions presented in the study are included in the article/Supplementary Material, further inquiries can be directed to the corresponding authors.
